# Nomogram based on the O-RADS for predicting the malignancy risk of adnexal masses with complex ultrasound morphology

**DOI:** 10.1186/s13048-023-01133-1

**Published:** 2023-03-21

**Authors:** Li-Ping Gong, Xiao-Ying Li, Ying-Nan Wu, Shuang Dong, Shuang Zhang, Ya-Nan Feng, Ya-Er Lv, Xi-Juan Guo, Yan-Qing Peng, Xiao-Shan Du, Jia-Wei Tian, Cong-Xin Sun, Li-Tao Sun

**Affiliations:** 1 Department of Ultrasound, Zhejiang Provincial People’s Hospital, Hangzhou, 310014 Zhejiang Province China; 2 Department of Ultrasound, The Second Affiliated Hospital of Harbin Medical University, Harbin, 150001 Heilongjiang Province China; 3 Department of Ultrasound, Shijiazhuang Obstetrics and Gynecology Hospital, Shijiazhuang, 050011 Hebei Province China

**Keywords:** Adnexal masses, Nomogram, O-RADS, CA125

## Abstract

**Objective:**

The accurate preoperative differentiation of benign and malignant adnexal masses, especially those with complex ultrasound morphology, remains a great challenge for junior sonographers. The purpose of this study was to develop and validate a nomogram based on the Ovarian-Adnexal Reporting and Data System (O-RADS) for predicting the malignancy risk of adnexal masses with complex ultrasound morphology.

**Methods:**

A total of 243 patients with data on adnexal masses with complex ultrasound morphology from January 2019 to December 2020 were selected to establish the training cohort, while 106 patients with data from January 2021 to December 2021 served as the validation cohort. Univariate and multivariate analyses were used to determine independent risk factors for malignant tumors in the training cohort. Subsequently, a predictive nomogram model was developed and validated in the validation cohort. The calibration, discrimination, and clinical net benefit of the nomogram model were assessed separately by calibration curves, receiver operating characteristic (ROC) curves, and decision curve analysis (DCA). Finally, we compared this model to the O-RADS.

**Results:**

The O-RADS category, an elevated CA125 level, acoustic shadowing and a papillary projection with color Doppler flow were the independent predictors and were incorporated into the nomogram model. The area under the ROC curve (AUC) of the nomogram model was 0.958 (95% CI, 0.932–0.984) in the training cohort. The specificity and sensitivity were 0.939 and 0.893, respectively. This nomogram also showed good discrimination in the validation cohort (AUC = 0.940, 95% CI, 0.899–0.981), with a sensitivity of 0.915 and specificity of 0.797. In addition, the nomogram model showed good calibration efficiency in both the training and validation cohorts. DCA indicated that the nomogram was clinically useful. Furthermore, the nomogram model had higher AUC and net benefit than the O-RADS.

**Conclusion:**

The nomogram based on the O-RADS showed a good predictive ability for the malignancy risk of adnexal masses with complex ultrasound morphology and could provide help for junior sonographers.

## Introduction

Ovarian cancer (OC) is one of the most aggressive and lethal malignancies. According to statistics, it has the highest mortality rate among all gynecological cancers and is often discovered at an advanced stage, with a 5-year survival rate of less than 30% [1]. The most common treatment for patients with initially diagnosed with OC is maximal debulking surgery followed by platinum-based adjuvant therapy [2]. In contrast, benign tumors can be managed with fertility conservation surgery or follow-up. Thus, accurate preoperative assessment is pivotal to the treatment and prognosis of OC.

Ultrasound (US) is the preferred imaging modality [3] for the preoperative evaluation of adnexal masses. Due to the varying characteristics in ultrasound images of adnexal masses and the dependence of operator experience, the accurate preoperative diagnosis of adnexal masses remains a great challenge for most junior sonographers, especially those with complex ultrasound morphology. At present, subjective assessment by the ultrasound experts remains the best method for the preoperative identification of adnexal masses [4, 5], but these experts are not always available.

A range of ultrasound-based predictive models and classification systems have been developed to help risk stratify adnexal masses [6–12]. In 2008, the International Ovarian Tumor Analysis (IOTA) group proposed the Simple Rules base on ten ultrasound features [8]. Nevertheless, the Simple Rules cannot be applied in all cases, as it classifies adnexal masses as benign, malignant and inconclusive, which limits its usefulness. The Gynecologic Imaging Reporting and Data System (GI-RADS) was developed in 2009 for the assessment of adnexal masses. However, it relies heavily on the subjective assessment of the sonographers rather than objective criteria, and is not universally accepted. The IOTA Assessment of Different Neoplasias in the Adnexa (ADNEX) model is the only model that can calculate the likelihood of multiple types of adnexal masses [10], including benign tumors, borderline tumors, stage I OC, stage II–IV OC, and metastatic cancer. Although the ADNEX model has high predictive value [13], it has not been widely used in North America and China to date. Recently, the American College of Radiology (ACR) released the Ovarian-Adnexal Reporting and Data System (O-RADS) ultrasound lexicon [14] and consensus guideline of risk stratification and management [12], which provide a standardized ultrasound lexicon for adnexal lesions and associated management schemes for all risk categories. The current studies showed that the O-RADS had a high diagnostic sensitivity but relatively low specificity [15–17], which means that the O-RADS can misdiagnose some benign masses as malignant and lead to overtreatment. Some scholars have proposed that the acoustic shadowing should be included in O-RADS classification system to improve its performance [18]. In addition, published studies have shown that the diagnostic performance of adnexal masses can be improved by combining ultrasound with clinical indicators [19, 20].

Therefore, we aimed to develop a model based on O-RADS in conjunction with other ultrasound and clinical indicators for predicting the malignancy risk of complex ultrasound morphology adnexal mass so as to improve the accuracy of junior sonographers.

## Materials and methods

### Patients

Data from women who underwent preoperative ultrasound examinations and surgery for adnexal masses at the Second Affiliated Hospital of Harbin Medical University between January 2019 and December 2021 were retrospectively analyzed. Patients from January 2019 to December 2020 were selected to establish the training cohort, while patients from January 2021 to December 2021 served as the validation cohort. All patients had complete ultrasound images and postoperative histological diagnoses. Borderline tumors were classified as malignant in this study due to their potential malignant biological behavior, susceptibility to recurrence, and potential for progression toward OC. The exclusion criteria were as follows: (a) ultrasound images of adnexal masses showing a unilocular cyst without solid component; (b) treatment before ultrasound examination; (c) a history of ovarian borderline tumor or OC; (d) an interval of more than 30 days between ultrasonography and surgery; (e) pregnancy.

### Data collection

Data on preoperative CA125 levels, age, menopausal status and postoperative histological diagnoses were collected for all patients. The postmenopausal state was defined as women who had been in amenorrhoea for more than 1 year and over the age of 50 years for those who had undergone hysterectomy or lacked records regarding menopause status. Elevated CA125 value was considered if > 35U/ml.

Most patients underwent transvaginal ultrasonography by experienced sonographers, and transabdominal sonography was additionally performed in patients whose adnexal masses were too large to be adequately assessed and in patiens for whom transvaginal ultrasound could not be performed for objective reasons. When multiple adnexal masses were detected in a patient, the lesion with the highest O-RADS category was included in this study. If the O-RADS categories were the same, the lesion with the largest maximum diameter was selected.

All ultrasound images were independently reviewed by two resident sonographers (with less than 3 years of gynecological ultrasound experience) who were blinded to the pathology findings. Before analyzing the images, the same two residents received theoretical training about the O-RADS lexicon [14] and risk stratification [12]. In this study, adnexal masses with complex ultrasound morphology included those lesions of multilocular cyst without solid component, unilocular cyst with solid component, multilocular cyst with solid component, and solid. According to the descriptor terms of the IOTA [21] and O-RADS strictly, the following ultrasound morphology features were recorded for each mass: maximum diameter of the lesion, maximum diameter of the largest solid component, internal margin or walls, external contour, number of locules, acoustic shadowing, number of papillary projections, papillary projection with color Doppler flow, vascularity, ascites. The degree of vascularity includes color score 1–4 according to the IOTA Group criteria [21], which represent no blood flow, minimal flow, moderate flow, and marked blood flow, respectively. The two sonographers categorized the adnexal masses separately by O-RADS risk stratification. If there was a disagreement between the two sonographers, all the details were discussed with the help of a senior sonographer until a consensus was reached.

### Statistical analysis

All analyses were performed using R version 4.2.1 and SPSS version 26.0. Continuous variables were described by medians (interquartile range [IQR]), and were compared by Mann–Whitney U test. Categorical variables were presented as numbers and percentages, and were compared using the chi-square test or Mann–Whitney U test. The interreviewer agreement (IRA) of two resident sonographers when using the O-RADS was evaluated by the kappa (κ) value. In the training cohort, univariate and multivariate analyses were used to determine risk factors and further develop a nomogram model that predicted the risk of malignancy of complex ultrasound morphology adnexal masses. Then, the model was verified in the validation cohort. The receiver operating characteristic (ROC) curve and area under the curve (AUC) were used to quantify the discriminative performance of the nomogram. Calibration curves and the Hosmer–Lemeshow test were used to evaluate the consistency of the model. Decision curve analysis (DCA) was conducted to assess the clinical usefulness of the nomogram by quantifying the net benefits. Furthermore, the sensitivity and specificity were calculated. The DeLong test was used to calculate the statistical significance of differences among the AUCs. Statistical significance was assumed at *p* < 0.05 for all comparisons.

## Results

### Patient characteristics

**Fig. 1:**
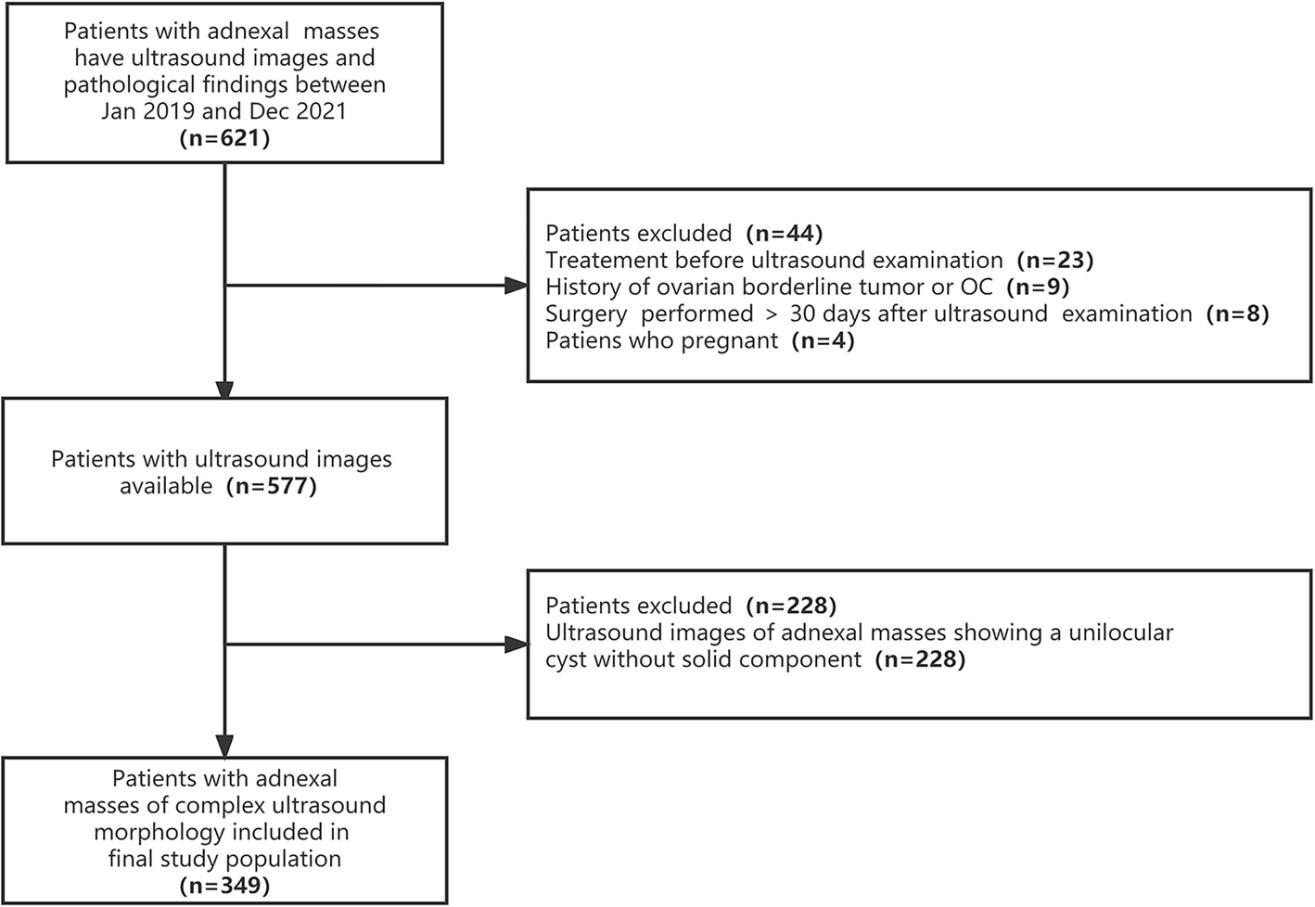
Flowchart of the study population

A total of 349 patients were recruited for this study (Fig. 1). Table 1 presents the detailed clinical and ultrasound characteristics for the training and validation cohorts, and the results showed that there were no significant differences between two cohorts (all *p* > 0.05). The malignancy rates of the training and validation cohorts were 53.9% (131/243) and 44.3% (47/106), respectively, with no significant difference (*p* = 0.100). Data comparisons between the benign and malignant groups in both cohorts showed that there were no significant differences in age or menopausal status (all *p* > 0.05). In addition to the characteristics of  > 3 papillary projections in the validation cohort (*p* = 0.059), there were obvious differences in ultrasound features between the benign and malignant groups, either within the training or validation cohorts.

**Table 1:** Clinical and ultrasound characteristics of patients with adnexal masses in the training and validation cohorts

**Characteristics**	**Training cohort**				**Validation cohort**				***P***^***c***^
	**All** **(*n* = 243)**	**Benign** **(*n* = 112)**	**Malignant** **(*n* = 131)**	***P***^***a***^	**All** **(*n* = 106)**	**Benign** **(*n* = 59)**	**Malignant** **(*n* = 47)**	***P***^***b***^	
Age (years, median (IQR))	50 (42–60)	50 (37–61)	49 (43–59)	0.882	49.5 (36–61)	45 (31–64)	50 (42–59)	0.696	0.641
Postmenopausal									
Yes	122	56	66	0.953	52	28	24	0.712	0.843
No	121	56	65		54	31	23		
CA125 > 35U/mL									
Yes	131	23	108	< 0.001*	50	12	38	< 0.001*	0.247
No	112	89	23		56	47	9		
O-RADS									
2	9	9	0	< 0.001*	4	4	0	< 0.001*	0.578
3	39	39	0		16	16	0		
4	105	59	46		52	36	16		
5	90	5	85		34	3	31		
Maximum diameter of the lesion (mm), median (IQR)	9.1 (6.2–12.3)	7.8 (5.1–11.1)	10.4 (6.8–13)	< 0.001*	8.6 (5–12.2)	7.9 (4.4–11.8)	8.6 (5.1–12.3)	< 0.001*	0.478
Maximum diameter of the largest solid component (mm), median (IQR)	3.8 (1.1–6.7)	1.4 (0–4.3)	5.0 (3.1–8.5)	< 0.001*	3.2 (1.3–5.4)	2.1 (0–3.6)	3.3 (1.3–5.4)	< 0.001*	0.167
Lesion category									
Multilocular cyst, no solid component	53	49	4	< 0.001*	23	22	1	< 0.001*	0.728
Unilocular cyst with solid component	48	12	36		25	6	19		
Multilocular cyst with solid component	60	8	52		21	7	14		
Solid	82	43	39		37	24	13		
Color score									
1	48	44	4	< 0.001*	26	23	3	< 0.001*	0.126
2	115	65	51		50	30	20		
3	48	2	46		25	6	19		
4	32	2	30		5	0	5		
Irregular internal wall	102	18	84	< 0.001*	50	17	33	< 0.001*	0.368
Irregular external contour	38	4	34	< 0.001*	11	1	10	0.003*	0.193
> 3 papillary projections	33	1	32	< 0.001*	7	1	6	0.059	0.060
Papillary projection with color Doppler flow	58	8	50	< 0.001*	18	4	14	0.002*	0.152
Acoustic shadowing	33	31	2	< 0.001*	24	23	1	< 0.001*	0.057
Ascites	28	2	26	< 0.001*	13	1	12	< 0.001*	0.843

*IQR* interquartile range, *O-RADS* Ovarian Adnexal Reporting and Data System, *CA 125* Cancer Antigen 125

^a^*P* value for univariate analysis of the training cohort; ^b^*P* value for univariate analysis of the validation cohort; ^c^*P* value for clinical characteristics analysis between the training and validation cohorts; ^*^ Indicates statistical significance

The IRA between the two sonographers when using the O-RADS was good (κ = 0.889, *p* < 0.001).

### Univariate and multivariate analyses

In the training cohort, six parameters were included to determine the risk factors for malignancy. In the univariate analysis, a higher category of O-RADS, elevated CA125 levels, the absence of acoustic shadowing, the presence of papillary projection with color Doppler flow, and a larger maximum diameter of the largest solid component were associated with malignant tumors (all *p* < 0.01, Table 2).

**Table 2:** Univariate and multivariate analyses of factors associated with malignancy

**Category**	**Univariate analysis**		**Multivariate analysis**	
	**χ**^**2**^** / T**	***P***	**OR**	***P***
Postmenopausal	0.004	0.953	__	__
O-RADS	10.903	< 0.001*	16.374	< 0.001*
CA125	93.129	< 0.001*	12.965	< 0.001*
Acoustic shadowing	35.186	< 0.001*	0.079	0.010*
Papillary projection with color Doppler flow	31.983	< 0.001*	4.559	0.007*
Maximum diameter of the largest solid component	6.986	< 0.001*	1.070	0.385

*O-RADS* Ovarian Adnexal Reporting and Data System, *CA 125* Cancer Antigen 125, *OR* Odds ratio

^*^Indicates statistical significance

Multivariable analysis showed that the O-RADS category, elevated CA125 levels, acoustic shadowing, and papillary projection with color Doppler flow were independent predictors for malignancy (all *p* < 0.05, Table 2).

### Nomogram to predict the risk of malignancy

**Fig. 2:**
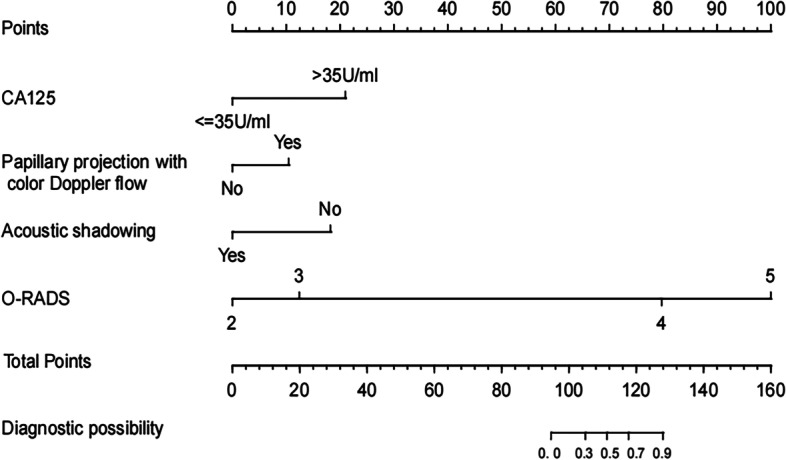
A nomogram for predicting the malignancy risk of adnexal masses with complex ultrasound morphology

**Fig. 3:**
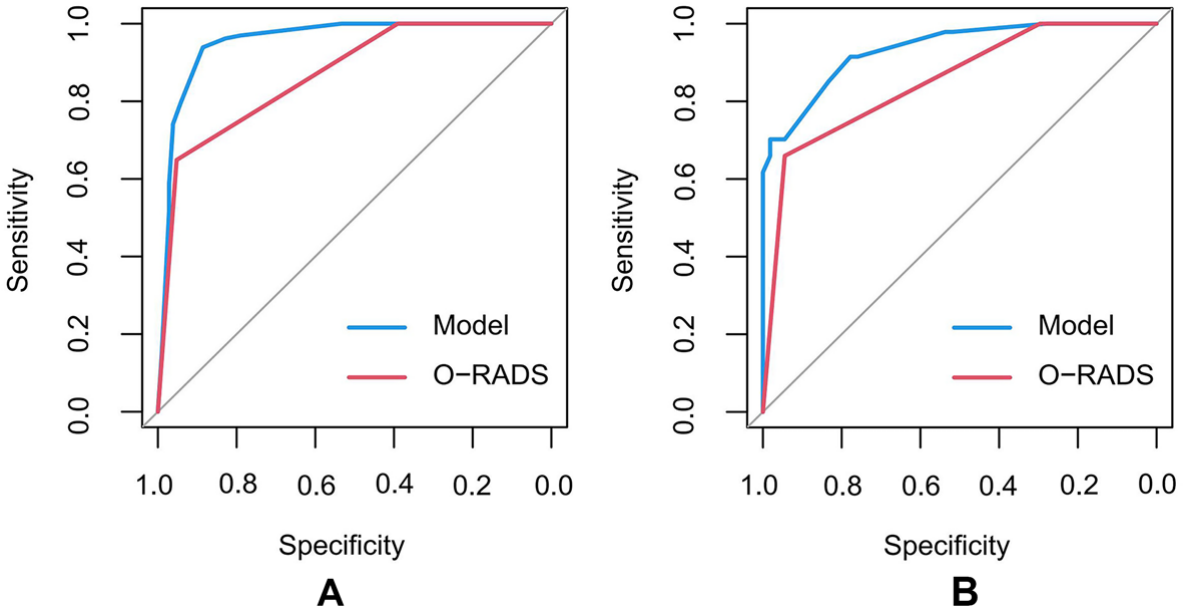
The ROC curves of the nomogram in each cohort. **A** The ROC curves in the training cohort. **B** The ROC curves in the validation cohort

**Fig. 4:**
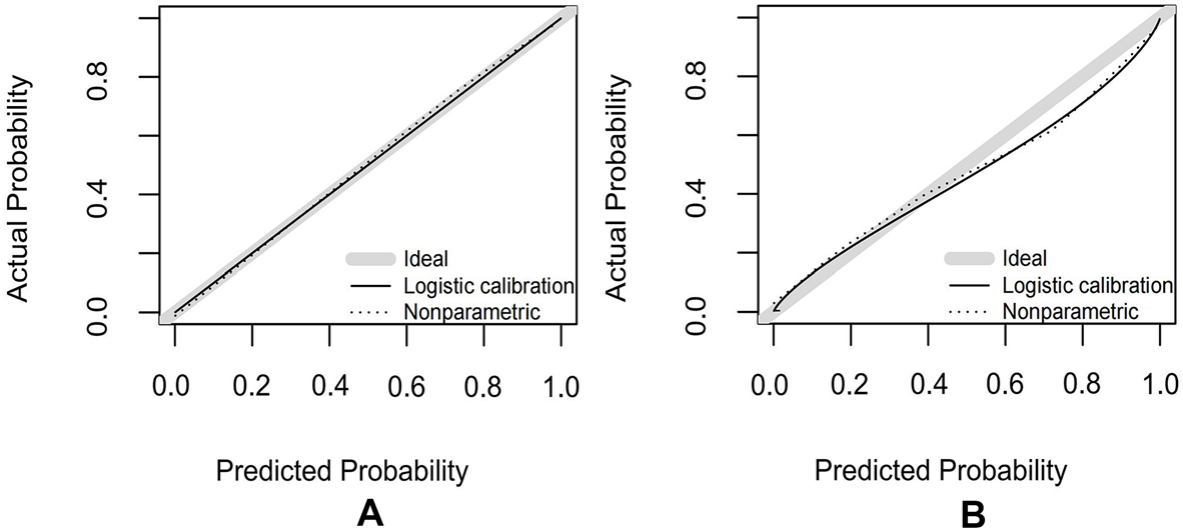
The calibration curves of the nomogram in each cohort. **A** The calibration curves in the training cohort. **B** The calibration curves in the validation cohort

According to the multivariate analysis, a nomogram incorporating O-RADS, CA125, acoustic shadowing, and papillary projection with color Doppler flow was constructed to predict the malignancy risk of adnexal masses with complex ultrasound morphology (Table 3) (Fig. 2). The nomogram showed that the O-RADS category was the most influential predictors of malignancy. ROC analysis in the training cohort showed that the AUC of the nomogram model was 0.958 (95% CI, 0.932–0.984), with a sensitivity of 0.939 and a specificity of 0.893 (Fig. 3). The calibration curves of the nomogram showed good consistency between the predicted probability of malignancy and the actual probability in the training cohort (Fig. 4), which was further supported by a non-significant result (*p* = 0.441) obtained by the Hosmer–Lemeshow test.

**Table 3:** Predictors of the malignancy risk of adnexal masses in the model

**Category**	**β**	**SE**	**OR**	**95% CI**	***P***
O-RADS	2.939	0.522	18.90	6.792–52.61	< 0.001*
CA125	2.788	0.495	16.26	6.17–42.86	< 0.001*
Acoustic shadowing	-2.338	0.986	0.097	0.014–0.667	0.018*
Papillary projection with color Doppler flow	1.451	0.561	4.266	1.421–12.803	0.010*

*O-RADS* Ovarian Adnexal Reporting and Data System, *CA 125* Cancer Antigen 125, *OR* Odds ratio

^*^Indicates statistical significance

### Validation of the nomogram

In the validation cohort, this model also exhibited a good AUC of 0.940 (95% CI, 0.899–0.981), with a sensitivity and specificity of 0.915 and 0.797 respectively (Fig. 3). Similarly, a good calibration of this model was observed in the validation cohort, and no significant difference was found by the Hosmer–Lemeshow test (*p* = 0.187) (Fig. 4).

### Clinical utility of the nomogram

**Fig. 5:**
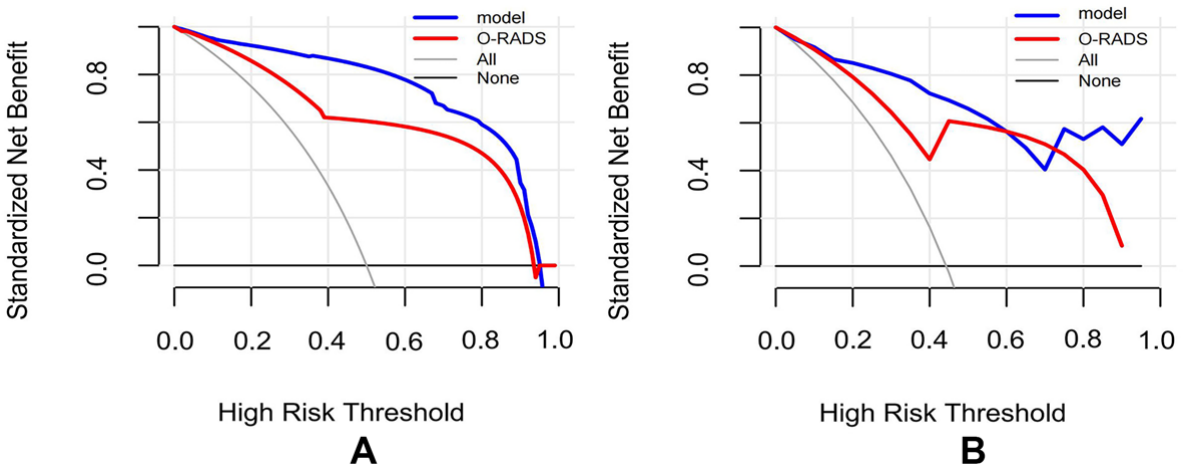
The DCA of the nomogram and the O-RADS in each cohort. **A** The DCA in the training cohort. **B** The DCA in the validation cohort

The DCA curves (Fig. 5) showed that clinical decisions based on the nomogram model had greater benefit than O-RADS in the training and validation cohorts, which suggested that the model may be used as an effective tool in clinical practice.

### Comparison of the nomogram to O-RADS

ROC curve (Fig. 3) analysis of O-RADS showed that O-RADS 5 was the best threshold for predicting malignancy risk in both the training and validation cohorts, indicating that the adnexal masses of O-RADS 5 were diagnosed as malignant and O-RADS 2–4 were diagnosed as benign. In this case, the AUC, sensitivity, and specificity of O-RADS in the training cohort were 0.877 (95% CI, 0.835–0.920), 0.649 and 0.955, respectively; those in the validation cohort were 0.862 (95% CI, 0.803–0.921), 0.660 and 0.949, respectively. However, when O-RADS 4 was used as the threshold, the AUC of O-RADS in the training cohort was only 0.714 (95% CI, 0.647–0.782), with a sensitivity of 1.00 and a specificity of 0.429; whereas those in the validation cohort were 0.669 (95% CI, 0.568–0.771), 1.00 and 0.339, respectively.

In contrast to O-RADS, the nomogram model exhibited better performance for malignancy prediction, with higher AUC and net benefit.

## Discussion

OC is the most fatal cancer among gynecological tumors, and seriously affects the life and health of women due to its low survival and high recurrence rates. Early detection, accurate diagnosis and referral to a gynecological oncologist are pivotal to the survival and prognosis of patients with OC, but these are still difficult for less experienced sonographers in their clinical work. In this study, we combined O-RADS with other ultrasonic indicators that included in other models or classification systems and clinical indicators to establish a model for predicting the malignancy risk of adnexal masses with complex ultrasound morphology. Finally, a nomogram consisting of O-RADS, CA125, acoustic shadowing, and papillary projection with color Doppler flow was obtained, and the results showed that the model had a favorable performance in predicting the malignancy risk of adnexal masses.

As the only standardized ultrasound lexicon for adnexal lesions and risk stratification system that includes all risk categories and related management schemes, the O-RADS has been widely studied by scholars since its release. A multicenter study [22] derived from 4905 masses for external validation of the O-RADS conducted that the O-RADS had 0.92 sensitivity and 0.80 specificity at the 10% risk threshold (O-RADS 4). A meta-analysis [15] involving 4634 adnexal masses from 11 studies showed that the sensitivity and specificity of the O-RADS were 0.97 and 0.77, respectively. Even though the overall diagnostic efficacy and sensitivity were good, relatively low specificity was still inevitable; in the study of Hiett et al., which only included 150 patients with adnexal masses and set the malignant risk threshold at 10% directly, the specificity was only 0.466 [23]. Furthermore, the specificity and sensitivity of O-RADS vary widely at different threshold risks [24, 25]. To date, there has been no consensus on the threshold of O-RADS in relation to the boundary between benign and malignant lesions or recommended surgery. Most studies have indicated that the optimal threshold for predicting malignancy was O-RADS 4 [23, 24, 26, 27], but in this study, it was O-RADS 5. Using the O-RADS 5 as a threshold for malignancy in this study, the AUC, sensitivity and specificity were 0.877, 0.649 and 0.955 respectively; whereas O-RADS 4 yielded a AUC only of 0.714, with a sensitivity of 1.00 and a specificity of 0.429. We speculated that the reason for this inconsistent optimal threshold and the poor performance at a threshold of O-RADS 4 was the different composition of study cohorts. The study cohort in our study was the adnexal masses with complex ultrasound morphology, which was different from other studies.

CA125 is the most widely used tumor biomarker for screening and monitoring epithelial ovarian cancer (EOC) [28]. However, it has always been controversial in clinical practice due to the high false-positive and false-negative rates. The United States Preventive Services Task Force also noted that isolated CA125 results were not recommended as an indicator for the diagnosis of OC [29]. In present study, the CA125 level in malignant tumors was significantly higher than that in benign tumors. As an independent risk factor for malignant tumors, CA125 had a high OR value (16.26) in the nomogram model, which indicated that CA125 had a high diagnostic value in adnexal masses with complex ultrasound morphology. Part of the reasons may be that most of the ovarian endometriosis cysts were excluded as unilocular cyst with solid component, thus the number was small in this study, which reduced the false-positive rate of CA125 to some extent. Acoustic shadowing often appears in benign adnexal masses, such as teratoma, cystadenofibroma, and fibroma [30, 31]. Acoustic shadowing was included in IOTA SR and ADNEX model as one of the key benign features [8, 10], but it has not been included in the O-RADS classification system. Studies have shown that adding acoustic shadowing to the O-RADS system can improve the diagnostic efficiency [22], and similar conclusion have been obtained in this study. The members of O-RADS US working group claimed that acoustic shadowing may appear in future iterations of the O-RADS [18]. As one of the malignant features in the LR model [6], in this study, the papillary projection with color Doppler flow mostly appeared in malignant tumors. Moreover, ultivariable analysis showed that the papillary projection with color Doppler flow was independent predictor for malignancy, which was consistent with previous studies [32, 33].

Previously, some scholars have also proposed to combine O-RADS with other ultrasound or serological indicators to improve diagnostic performance. Wang et al. [34] proposed a simple combination of O-RADS, HE4 and CA125, in which an adnexl mass was diagnosed as benign if all three results were negative; otherwise, it is diagnosed as malignant. However, endometriosis cysts with elevated CA125 were misdiagnosed as malignant using this method. A recent study [35] indicated that a combination of O-RADS, SR and CA125 had a significantly higher AUC in discriminating ovarian tumors than individual approaches, but it did not provide the specific method. Wu et al. [36] developed a model of combining O-RADS and CA125, and the results suggested that the model significantly increased the diagnostic performance of malignancy risk estimation in adnexal masses. In addition, the model also showed good results in distinguishing certain subtypes of ovarian tumors. However, they only provided the mathematical formula of the model, and did not conduct a comprehensive evaluation and verification of the model.

In clinical practice, sonographers are usually able to correctly diagnose the adnexal masses with simple morphology, such as unilocular cysts without solid component. As previously reported, the risk of malignancy of the unilocular cysts is less than 1% [37, 38]. However, it is more difficult to distinguish the adnexal masses with complex ultrasound morphology, especially for junior sonographers. Therefore, this study excluded the adnexal masses of unilocular cysts without solid component and attempted to develop a nomogram for predicting the malignancy risk of adnexal masses with complex ultrasound morphology. The results showed that the model presented in this study had high predictive efficiency in the training cohort (AUC = 0.958) and validation cohort (AUC = 0.940), and had a superior performance than the O-RADS. These findings indicated that this model can help junior sonographers to identify adnexal masses and improve their confidence.

One of limitations of the present study was its retrospective nature that all ultrasound images were static and stored with varied quality, which may have influenced the assessments of the sonographers. Additionally, only patients who had undergone gynecological surgery were included in this study, which may cause selection bias. Finally, the model was based on data from a single tertiary center and the small sample may not be representative, and the applicability of this model still requires external validation by additional databases from other regions and countries.

## Conclusion

In summary, the results suggested the nomogram based on the O-RADS showed a better predictive ability than O-RADS for the malignancy risk of adnexal masses with complex ultrasound morphology. This nomogram may hold potential values in helping junior sonographers identify adnexal masses.

## Data Availability

All data generated or analyzed during this study are included in this published article.
